# miR-26b Promotes Granulosa Cell Apoptosis by Targeting *ATM* during Follicular Atresia in Porcine Ovary

**DOI:** 10.1371/journal.pone.0038640

**Published:** 2012-06-21

**Authors:** Fei Lin, Ran Li, Zeng xiang Pan, Bo Zhou, De bing Yu, Xu guang Wang, Xue shan Ma, Jing Han, Ming Shen, Hong lin Liu

**Affiliations:** Department of Animal Genetics, Breeding and Reproduction, College of Animal Science and Technology, Nanjing Agricultural University, Nanjing, China; VU University Medical Center, The Netherlands

## Abstract

More than 99% of ovarian follicles undergo atresia in mammals, but the mechanism of follicular atresia remains to be elucidated. In this study, we explored microRNA (miRNA) regulation of follicular atresia in porcine ovary. A miRNA expression profile was constructed for healthy, early atretic, and progressively atretic follicles, and the differentially expressed miRNAs were selected and analyzed. We found that miR-26b, which was upregulated during follicular atresia, increased the number of DNA breaks and promoted granulosa cell apoptosis by targeting the ataxia telangiectasia mutated gene directly *in vitro*.

## Introduction

Only a limited number of follicles develop to ovulation in mammals, and more than 99% of ovarian follicles undergo degeneration known as “follicular atresia” at any stage of growth and development [1], [2]. Atresia is a key process that occurs in the ovary to eliminate follicles that will not ovulate. Degeneration of atretic follicles can be explained partially by apoptosis of granulosa cells, theca interna cells, and oocytes, which exhibit a condensed nuclear structure, compacted cytoplasmic organelles, decreased cell size, and DNA fragmentation [3], [4]. A recent study indicated that follicular atresia is triggered by granulosa cell apoptosis [5], but the actual molecular mechanism of follicular atresia is still unknown.

MicroRNAs (miRNAs) are endogenous small noncoding RNAs of approximately 22 nucleotides in length [6] that can partially negatively regulate gene expression at posttranscriptional level by degrading or deadenylating target mRNA or by inhibiting translation. miRNA sequences are highly conserved between species. Since lin-4 and let-7 were found respectively in *Caenorhabditis elegans* in 1993 and 2000 [7], [8], numerous miRNAs have been identified in many animals, plants, and viruses (http://www.mirbase.org/) [9], [10]. miRNAs play important roles in a large variety of biological and cellular processes such as development, differentiation, proliferation, apoptosis, and tumorigenesis [11], [12], [13], but the mechanism of miRNA regulation has yet to be elucidated. Although miRNAs have been demonstrated to take part in a wide range of physiological process, no data on the relationship between miRNAs and follicular atresia have been reported until now.

Many studies have been conducted on follicular atresia in pigs, and many factors are reported to take part in this process such as tumor necrosis factor (TNF) [14], TNF-related apoptosis-inducing ligand [15], Fas ligand [16], X-linked inhibitor of apoptosis protein (XIAP) [17]. However, whether miRNA, an important epigenetic strategy to regulate gene expression, is applicable to the regulation of pig follicle atresia is unclear. In this study, we evaluated miRNA expression in porcine ovarian follicles using a miRNA microarray assay. Dozens of differentially expressed miRNAs were identified, and the target genes and the functions of these miRNAs were predicted. Then, we focused on the differential expression of miR-26b, which was upregulated during atresia. We found that miR-26b could induce pig granulosa cell apoptosis *in vitro*, that ataxia telangiectasia mutated (*ATM*) was a direct target gene of miR-26b, and that DNA breaks increased in granulosa cells transfected with miR-26b.

## Results

### miRNA Expression Profiles During Follicular Atresia in Porcine Ovary

**Figure 1:**
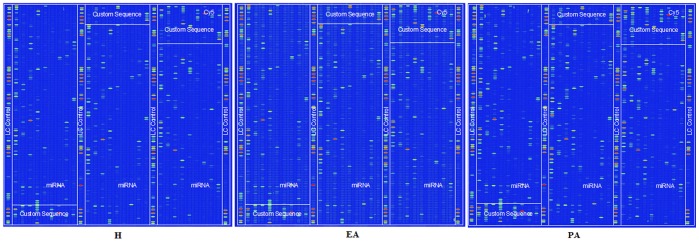
miRNA expression profile during porcine follicular atresia. Healthy (H), early atretic (EA), progressively atretic (PA) are representative regions of the Cy5 chip images. The images are displayed in pseudo-colors to expand the visual dynamic range. As the signal intensity increased from 1 to 65,535, the corresponding color changed from blue to green to yellow and red.

The µParaflo™ miRNA microarray assay was used to evaluate the expression profiles of 1251 mature miRNAs during pig follicular atresia (Figure 1). They were composed of three parts: 1053 unique mature miRNAs from pig, human, and mouse (based on Sanger miRBase release 13.0, marked by species); 99 miRNA sequences from references (marked by R) [18], [19], [20]; and 99 pig miRNA candidates predicted in our laboratory (marked by P) [21]. In total, 329, 369, and 435 miRNAs were detected in the healthy (H), early atretic (EA), and progressively atretic (PA) follicles, respectively. Nearly 200 differentially expressed miRNAs (*P*<0.01) were identified by ANOVA. The EA/H value was used for further screening because we focused on the initiation of follicular atresia. 23 miRNAs (at least one signal value >1000 with an EA/H >2 or an EA/H <0.7) were selected (Table 1), 12 of which were upregulated and 11 of which were downregulated.

**Table 1:** Differentially expressed miRNAs during porcine follicle atresia.

miRNA	H Mean	EA Mean	PA Mean	EA/PA	p-value
Upregulated miRNAs					
hsa-miR-936	319.67	1282.51	1003.06	4.01	1.70526E−05
P-miR-1281	464.38	1675.19	2167.30	3.61	3.70351E−06
hsa-miR-26b	172.13	561.54	1623.17	3.26	1.40894E−06
mmu-miR-1224	945.32	3012.84	2676.98	3.19	0.00056122
hsa-miR-10b	723.69	1760.22	2311.29	2.43	8.94694E−06
P-miR-466g-b	1205.42	2842.93	1363.18	2.36	0.002424305
P-miR-1275	1396.46	3030.95	1666.71	2.17	0.00172605
hsa-miR-574-5p	640.70	1375.41	293.17	2.15	0.000333658
R-miR-26b	1803.18	3869.54	9927.83	2.15	3.80023E−05
hsa-miR-149*	488.54	1025.32	1320.74	2.10	0.003760175
hsa-miR-1275	1414.95	2912.71	2052.27	2.06	0.000699006
hsa-miR-99a	353.72	708.64	1956.51	2.00	3.57687E−05
Downregulated miRNAs					
R-let-7a	41,704.41	29,099.82	15,432.83	0.70	1.18518E−05
hsa-let-7i	6930.18	4812.24	3630.25	0.69	0.000108401
hsa-miR-92b	910.77	613.02	658.31	0.67	0.002838088
hsa-miR-92a	6692.46	4483.72	2836.03	0.67	4.37391E−06
P-miR-923	37,929.77	25,246.05	13,029.16	0.67	1.67734E−07
hsa-miR-1979	32,600.93	19,093.57	8068.98	0.59	5.4785E−07
R-miR-739	43,558.83	22,871.56	11,358.65	0.53	2.24742E−06
hsa-miR-1308	7470.44	3576.30	2243.58	0.48	6.97377E−07
hsa-miR-1826	68,631.36	31,756.20	17,899.43	0.46	5.60287E−07
P-miR-1826	125,609.86	51,011.88	26,731.74	0.41	1.24318E−08
ssc-miR-184	1395.88	517.12	91.29	0.37	2.89758E−07

H, healthy; EA, early atretic; PA, progressively atretic.

### Target Genes and Functions of Differentially Expressed miRNAs

Target genes were used to predict the functions of these differentially expressed miRNAs. After the GO and Pathway analysis, 324 significant target genes (Figure S1) were selected with widespread roles in cell processes such as cell proliferation, differentiation, adhesion, apoptosis, and DNA replication (Figure S2). Let-7a and miR-26b, with target gene numbers 83 and 73 respectively, were the two most prevalent miRNAs. Seven miRNAs (let-7a, miR-26b, miR-10b, miR-1275, miR-1308, miR-1826, and miR-936) seemed to be involved in apoptosis. The miRNAs and their target genes included in apoptosis pathway are shown in Table 2. miR-26b was upregulated during follicular atresia with many predicted target genes, and may be involved in apoptosis based on the miRNA-GO and miRNA-Pathway analysis. Moreover, miR-26b suppresses cell growth and induces cell apoptosis *in vitro* [22], so it was chosen for further research.

**Table 2:** miRNAs involved in the apoptosis pathway.

microRNAs	Target genes	Pathway name
R-let-7a	BCL2L1	Apoptosis
R-let-7a	CASP3	Apoptosis
R-let-7a	PPP3CA	Apoptosis
R-let-7a	TNFSF6	Apoptosis
hsa-miR-26b	*ATM*	Apoptosis
hsa-miR-26b	PPP3R1	Apoptosis
hsa-miR-1826	NFKB1	Apoptosis
hsa-miR-1826	XIAP	Apoptosis
hsa-miR-1308	BCL2	Apoptosis
hsa-miR-936	CAD	Apoptosis

### miR-26b was Upregulated During Follicular Atresia

**Figure 2:**
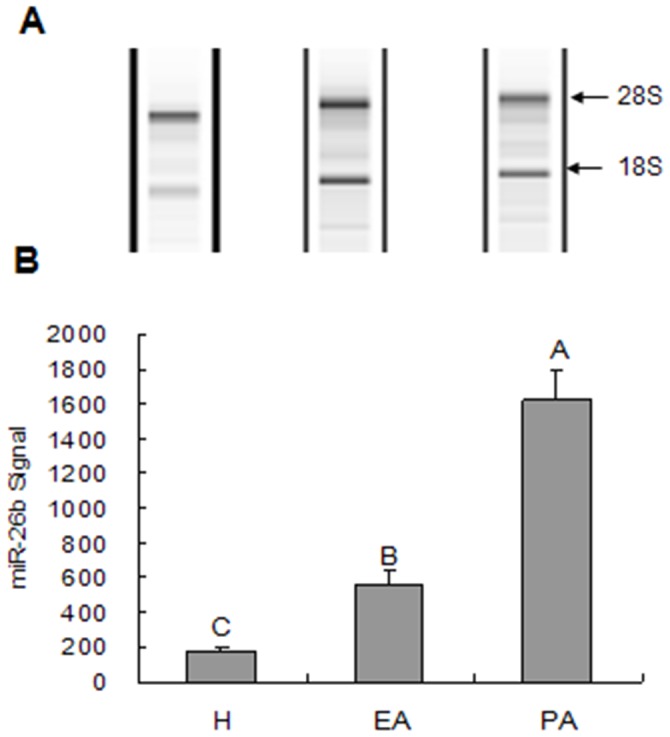
miR-26b expression during follicular atresia. miR-26b expression was detected by miRNA microarray assay. A, The quantity of RNA used in the microarray assay, and the integrity of 18 s and 28 s rRNA was checked. B, miR-26b signals were detected in healthy (H), early atretic (EA), and progressively atretic (PA) follicles. miR-26b expression was upregulated during porcine follicular atresia.

We first focused on the expression level of mature miR-26b in H, EA, and PA follicles. The miRNA array assay showed that miR-26b was upregulated during follicular atresia in porcine ovarian follicles (Figure 2).

### miR-26b Promotes Granulosa Cell Apoptosis in Vitro

**Figure 3:**
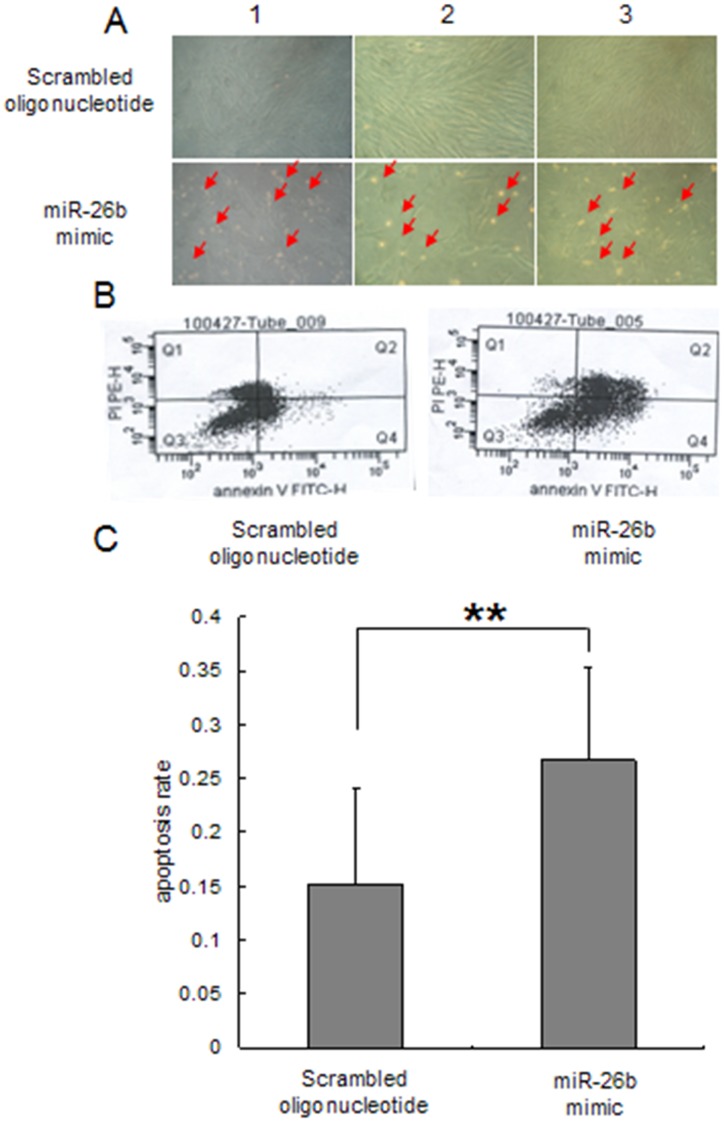
The effect of miR-26b on granulosa cell apoptosis *in vitro*. A, Porcine granulosa cells undergo apoptosis after miR-26b mimic treatment (three replicates). Granulosa cells were transfected with the miR-26b mimic or scrambled oligonucleotide (150 nM) after an additional 72-h culture (red arrow shows apoptosis cells). B and C, flow cytometry analysis (FACS) of apoptosis. After treatment and additional culture, cells were harvested and stained with Anti-annexin V-propidium iodide followed by FACS analysis. Apoptosis improved significantly following the miR-26b transfection.

Granulosa cell apoptosis is involved in follicular atresia [5], [23]. We verified the effect of miR-26b on cultured granulosa cell *in vitro* to investigate the role of miR-26b during follicular atresia. Porcine granulosa cells were transfected with the miR-26b mimic or scrambled oligonucleotide. After a 72-h incubation, the cells transfected with the miR-26b mimic died by apoptosis and were floating in the medium; however, cells transfected with the scrambled oligonucleotide grew well (Figure 3A). Anti-annexin V-propidium iodide (PI) staining and FACS analysis confirmed that cell apoptosis increased significantly after transfection of the miR-26b mimic (Figure 3B and C). These data suggest that miR-26b promotes granulosa cell apoptosis.

### ATM is a Target of miR-26b

**Figure 4:**
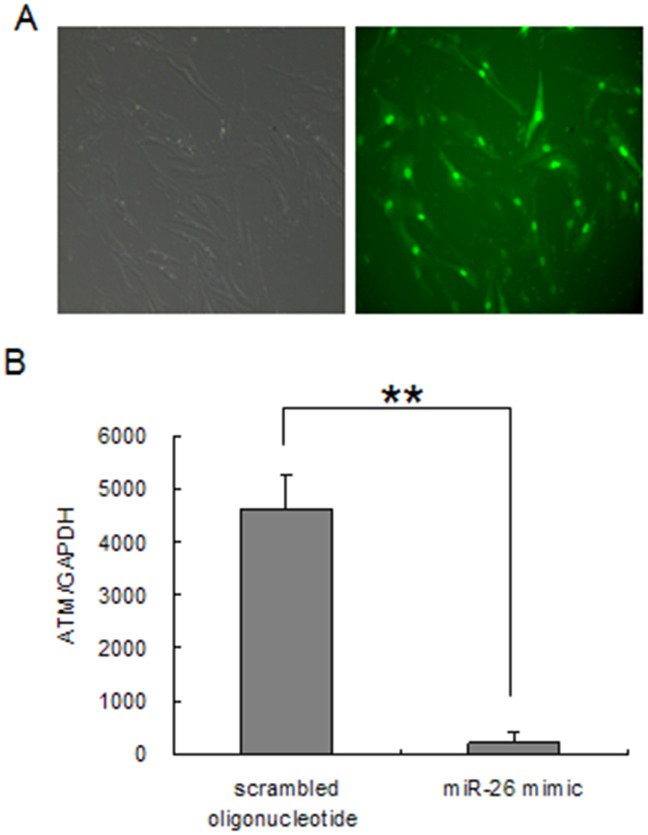
Regulation of ataxia telangiectasia mutated gene (*ATM*) expression by miR-26b. A, Transfection efficiency. Granulosa cells were transfected with a scrambled oligonucleotide marked with FAM (150 nM), and fluorescence was checked within 12 h after transfection. B, *ATM* expression was downregulated by miR-26b. Granulosa cells were transfected with scrambled oligonucleotide or an miR-26b mimic, and *ATM* expression was checked by real-time PCR (**P<0.01 compared to the scrambled oligonucleotide).

*ATM* may be a target gene for miR-26b based on a bioinformatics analysis. To focus on *ATM* expression after miR-26b mimic treatment, we transfected porcine granulosa cells with the miR-26b mimic, scrambled oligonucleotide, or scrambled oligonucleotide marked with FAM to reflect transfection efficiency (>90%) (Figure 4A). After a 72-h incubation, we checked *ATM* expression by real-time PCR and found that the miR-26b mimic decreased *ATM* expression at the mRNA level (Figure 4B).

**Figure 5:**
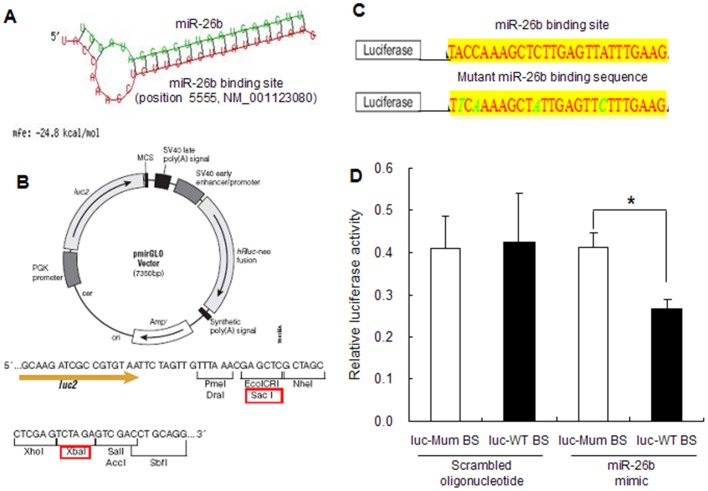
miR-26b binding site within *ATM* mRNA mediates downregulation of *ATM* by miR-26b. A, A miR-26b binding site within *ATM* mRNA was predicted by RNAhybrid. B, Map of the pmirGLO luciferase reporter vector. The red rectangles show the restrictive endonuclease used for cloning. C, miR-26b binding sequence for the wild type and mutant (shown in green). D, Luciferase activity assay. Granulosa cells were transfected with luciferase vectors including the wild-type or mutant miR-26b binding site and the miR-26b mimic or a scrambled oligonucleotide and harvested for the dual luciferase reporter assay after 24 h. BS, binding site.

While exploring the mechanism for the downregulated *ATM* expression by miR-26b, a potential binding site for miR-26b was found on the *ATM* mRNA at position 5555 (NM_001123080) (Figure 5A). We hypothesized that miR-26b represses *ATM* expression through this site, so we constructed two reporter vectors including luciferase cDNA followed by an miR-26b binding site or mutant miR-26b binding site (Figure 5B and C) as a control. The reporter vectors were co-transfected into granulosa cells with miR-26b mimic or scrambled oligonucleotide. miR-26b decreased luciferase activity of the reporter vector containing the wild-type binding site, but not the reporter with the mutant response element. In contrast, the scrambled oligonucleotide had no effect on luciferase activity of the wild-type reporter or the mutant vector (Figure 5D). Taken together, miR-26b inhibited *ATM* expression directly by binding to its mRNA at position 5555.

### DNA Break Increased after miR-26b Transfection

**Figure 6:**
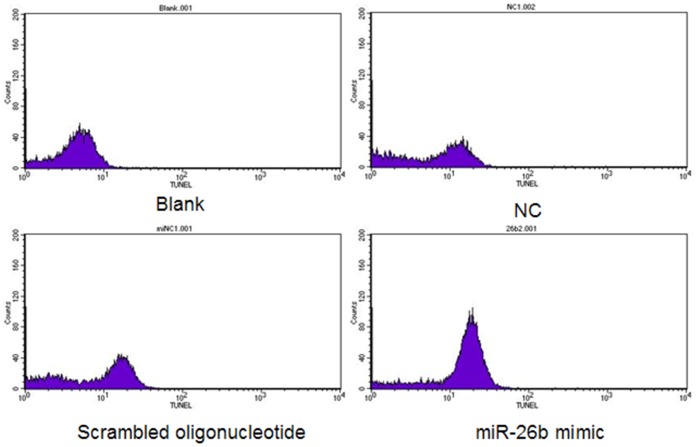
DNA breaks analysis by terminal deoxyribonucleotidyl transferase-mediated dUTP nick end labeling (TUNEL) assay. Granulosa cells were transfected with the miR-26b mimic or a scrambled oligonucleotide (150 nM) after an additional 72-h incubation. Cells were harvested for TUNEL stain and flow cytometry analysis. Blank, cells not transfected and without TUNEL stain; NC, cells not transfected.

*ATM* plays an important role in DNA repair [24]. If miR-26b promotes cell apoptosis by targeting *ATM*, then DNA breaks must increase after miR-26b mimic transfection. To explore the effect of miR-26b on DNA breaks, we performed the TUNEL (Terminal-deoxynucleoitidyl Transferase Mediated Nick End Labeling) assay in porcine granulosa cells cultured *in vitro*. Granulosa cells with DNA breaks increased significantly in the miR-26b mimic transfection group (Figure 6). Therefore, miR-26b induced an increase in the number of DNA breaks in porcine granulosa cells *in vitro*.

## Discussion

Gonadotropins [25], gonadal hormones [26], [27], growth factors [28], cell adhesion molecules [29], and cell death ligands and receptors [5] are involved in follicular atresia. Our results showed that miRNA, an important epigenetic strategy to regulate gene expression, also contributed to follicular atresia. Because known porcine miRNAs are rare, we performed a miRNA microarray assay of human, mouse, and predicted porcine miRNA candidates to evaluate the expression of miRNAs during follicular atresia in pigs. miRNA expression profiles have been reported for fibroblast cells [30], muscle and adipose tissue [31], and the intestine of pigs [32], however, we conducted a miRNA microarray assay in follicles, and more than 400 miRNAs were expressed in the pig ovary. 23 differentially expressed miRNAs that may be associated with atresia were identified, the functions of these differentially expressed miRNAs remain to be studied. Although the majority was not accepted in porcine by miRBase 13.0, homologous porcine miRNAs were identified and are included in miRBase 16.0. These miRNAs were miR-1224, miR-10b, miR-574, miR-99a, let-7a, let-7i, miR-92a, and miR-92b. let-7a suppressed cancer cell death by targeting caspase-3 [33] and suppressing miR-184 could induce apoptosis [34]. These results agreed with our data, which made our result believable.

Porcine genes are highly homologous to humans, so we performed a target gene scan for miRNA in the human genome. Pro-survival and pro-apoptotic molecules are involved in ovarian apoptosis [35] and a delicate balance between them determines the final destiny of follicular cells [36]. In this study, hundreds of target genes were pretested, tens of which were involved in apoptosis, including pro-apoptosis genes (such as *CASP3* and *TNFSF6*) and anti-apoptosis genes (such as *BCL2* and *XIAP*). This suggested that miRNAs are involved in the regulation of pro-survival and pro-apoptotic gene balance. One miRNA can target numerous genes, and one gene can be regulated by several miRNAs. Our data showed that let-7a and miR-26b targeted as many as 83 and 73 genes, respectively, and that nemo-like kinase was simultaneously regulated by let-7a, miR-26b, miR-92a, and miR-936. More attention should been paid to miRNAs with more apoptosis-related target genes, including let-7a, miR-26b, mir-1826, and miR-936. These miRNAs are more likely to play roles during follicular atresia, but their functions remain to be determined.

Although miR-26b has not been accepted in porcine by miRBase 16.0 until now, miR-26b is expressed in pig skeletal muscles and is found in RNA pools from the heart, thymus, and liver [20], [37]. Our results suggested that miR-26b was also expressed in porcine ovary and was upregulated during follicular atresia. The function of miR-26b is unclear because few studies have investigated this miRNA. miR-26b regulates pituitary development by targeting lymphoid enhancer factor 1 [38] and is downregulated in cancers [39], [40]. miR-26b suppresses cell growth, induces apoptosis *in vitro* [22], and inhibits cell proliferation by regulating cyclooxygenase-2 expression [41]. Our results demonstrated that miR-26b promotes porcine granulosa cell apoptosis by targeting a member protein of the phosphatidylinositol-3-kinase-like enzyme family [42], *ATM*, which is mutated in the human disease ataxia telangiectasia.

Although the apoptosis rate improved significantly after miR-26b mimic transfection, it was not upregulated as much as we expected. This may be due to the observation that DNA breaks occur naturally without genotoxic agent treatment, as in this study. The other reasonable explain of this phenomenon is that *ATM* was downregulated by miR-26b, and that residual *ATM* may still have been expressed in granulosa cells. We demonstrated that miR-26b downregulated *ATM* directly and that reduced *ATM* expression induced granulosa cell apoptosis *in vitro*. An associated *in vivo* study was conducted using *ATM* knockout as early as 1996. *ATM*-deficient mice were infertile, and ovaries from mutant females were devoid of primordial and maturing follicles and oocytes [43], indicating that an *ATM* deficiency not only promotes apoptosis in granulosa cells but also in oocytes, as *ATM* plays an important role in DNA repair [24]. In agreement with this result, the pathologies associated with ATM disease are attributable largely to defective DNA double-strand break recognition and repair [44].Thus, DNA breaks increased greatly after the miR-26b mimic transfection.

In conclusion, a miRNA expression profile was constructed during porcine follicular atresia, and candidate miRNAs regulating follicle atresia were chosen. miR-26b increased the number of DNA breaks by targeting the *ATM* gene and promoting porcine granulosa cell apoptosis *in vitro*.

## Materials and Methods

### Ethics Statement

All experiments were performed according to Nanjing Agricultural University Animal Care and Use Committee guidelines and all animal work was approved by the committee. All pigs were killed in a state of unconsciousness, and then ovaries were taken.

### Animal and Follicle Separation

Porcine ovaries were obtained from mature sows at a local slaughterhouse (Tianhuan Company) and transferred to the laboratory as soon as possible in physiological saline at 30°C–35°C. Individual preovulatory antral follicles (3–5 mm in diameter) were dissected from the ovaries under a surgical dissecting microscope (SZ40; Olympus, Tokyo, Japan) with small scissors and forceps. Each follicle was classified morphologically as healthy (H), early atretic (EA), or progressively atretic (PA) [17], [45]. Briefly, healthy follicles were round with a sharp and continuous granulosa cell membrane; they had a fixed and visible cumulus–oocyte complex [46] and clear follicular fluid. Early atretic follicles may still have a visible COC but with gaps in membrane granulosa cells and turbid follicular fluid. PA follicles did not have a visible COC or had a COC in follicular fluid with dark floccules [47], [48].

### Chemiluminescence for 17β-estradiol (E2) and Progesterone (P4) Levels in Follicular Fluid

Follicular fluid was collected and centrifuged at 3000 × g for 5 min to separate granulosa cells. An aliquot of each sample was diluted 100-fold with double-distilled water. The diluted fluid was used to quantify E_2_ and P_4_ levels using E_2_ and P_4_ kits (Beckman, Fullerton, CA, USA), respectively, at the General Hospital of the Nanjing Military Command. The P_4_/E_2_ (P/E) value was used to confirm the follicle classification [49]. The E_2_ and P_4_ levels in follicles selected for the miRNA microarray assay are shown in Table 3. Only follicles with P/E values in accordance with morphology were selected.

**Table 3:** Follicle information used in the miRNA microarray assay.

	Follicle number	P(pg/ml)	E(pg/ml)	P/E	Diameter (mm)	Morphology
H	1	1091.2	886.3	1.2	5.0	H
	2	1333.4	816.6	1.6	4.5	H
	3	1487.4	1016.5	1.5	4.0	H
	4	3824.0	1871.7	2.0	5.0	H
	5	2493.7	1187.8	2.1	4.0	H
EA	1	987.4	119.3	8.3	5.2	EA
	2	1195.0	121.5	9.8	3.2	EA
	3	1116.4	135.6	8.2	5.0	EA
	4	1292.5	179.5	7.2	3.0	EA
PA	1	1220.1	86.1	14.2	4.5	PA
	2	1440.3	114.1	12.6	4.5	PA
	3	1940.3	73.8	26.3	4.0	PA
	4	2091.2	75.2	27.8	5.3	PA
	5	1081.8	84.2	12.9	4.0	PA

### µParaflo™ MicroRNA Microarray Assay

The microarray assay was conducted using a service provider (LC Sciences, Houston, TX, USA). The assay used from 2 to 5 µg of total RNA. Hybridization was performed overnight on a µParaflo™ microfluidic chip using a micro-circulation pump [50], [51]. The hybridization used 100 µl 6× SSPE buffer (0.90 M NaCl, 60 mM Na_2_HPO_4_, 6 mM EDTA, pH 6.8) containing 25% formamide at 34°C. Hybridization images were collected and digitized. Data were analyzed by first subtracting the background and then normalizing the signals using a locally weighted regression filter [52].

### Potential Targets and Functions of Differentially Expressed miRNAs

Target gene, GO, and Pathway analyses were performed by the Shanghai Qiming Information Technology Company. (Shanghai, China). After targeted gene prediction, the GO analysis was applied to analyze the main function of the differentially expressed miRNAs according to Gene Ontology, which is the key functional classification of NCBI. Pathway analysis was used to identify the significant differential gene pathways, according to KEGG, Biocarta, and Reatome.

### Granulosa Cell Culture and miRNA Transfection

Granulosa cells were collected from porcine ovaries using a syringe; they were washed with PBS and cultured in DMEM/F12 (1∶1) with 10% fetal bovine serum in an incubator with 5% CO_2_ in air at 37°C. Penicillin (100 units/ml) and streptomycin (100 µg/ml) were used in the cultures. A miR-26b mimic and scrambled oligonucleotides were obtained from GenePharma (Shanghai, China). Granulosa cells were transfected with the miR-26b mimic or scrambled oligonucleotide (150 nM) for 6 h using Lipofectamine 2000. All experiments were repeated in triplicate.

### Apoptosis Analysis

Granulosa cells were transfected with the miR-26b mimic or scrambled oligonucleotide as a control. After an additional 72-h incubation, the cells were harvested, stained with propidium iodide and anti-annexin V-FITC, and analyzed by flow cytometry to determine the relative amount of AnnexinV-FITC positive and PI-negative cells.

### Real-time PCR

Total RNA was extracted using TRIZOL, and the RT reactions were performed with MLV, according to the manufacturer’s protocol. Real-time PCR was amplified with SYBR Premix Ex Taq in a reaction volume of 20 µl. Primer sequences were as follows: *ATM*, forward primer 5′-GGCTGTCACTGATAGAGGG-3′ and reverse primer 5′-AAGGCAACTTAGGGTAGGA-3′, 243 bp; GAPDH, forward primer 5′-GGACTCATGACCACGGTCCAT-3′ and reverse primer 5′-TCAGATCCAC AACCGACACGT-3′, 220 bp.

### Plasmid Construction

The predicted miR-26b binding site (italics), with the *Sac*I*/Xba*I enzyme site and the sequence CTAGGATCCCTTA*TACCAAAGCTCTTGAGTTATTTGAAG*ATAAT was synthesized, reannealled, and cloned into the *Sac*I*/Xba*I site of the pmirGLO luciferase vector. The pmiR-GLO luciferase vector contained the mutant miR-26b binding site, whose sequence was CTAGGATCCCTTA*T* ***T*** *C* ***A*** *AAAGCT* ***A*** *TTGAGTT* ***C*** *TTTGAAG*ATAAT (the four italic and bold nucleotides are mutated); it was constructed simultaneously.

### Luciferase Assay

Granulosa cells were plated in 6-well plates. The next day, 1000 ng/ml pmirGLO luciferase vector, including the wild-type or mutant miR-26b binding site and 50 pmol/ml miR-26b mimic or scrambled oligonucleotide, was transfected using Lipofectamine 2000. Luciferase assays were performed using the dual luciferase reporter assay system 24 h after transfection.

### TUNEL Assay

The terminal deoxyribonucleotidyl transferase mediated dUTP nick end labeling (TUNEL) method, which examines DNA strand breaks, was conducted with the In Situ Cell Death Detection Kit (Roche Applied Science, Shanghai, China). Flow cytometry was also performed to detect DNA breaks in cells.

### Statistical Analysis

All results are expressed as means ± SE. The statistical analysis was performed with a *t*-test to compare two groups, and an analysis of variance (ANOVA) was applied for multiple comparisons. A *P*<0.05 was considered statistically significant.

## Supporting Information

Figure S1 **Potential targets prediction for differential expression miRNAs.**
(TIF)

Figure S2 **GO anslysis of potential targets.**
(TIF)
