# Supplementary figures and images for: miR-26b Promotes Granulosa Cell Apoptosis by Targeting ATM during Follicular Atresia in Porcine Ovary
Source: PLoS One. 2012 Jun 21;7(6):e38640. doi: 10.1371/journal.pone.0038640 (PMC3380909; doi:10.1371/journal.pone.0038640)

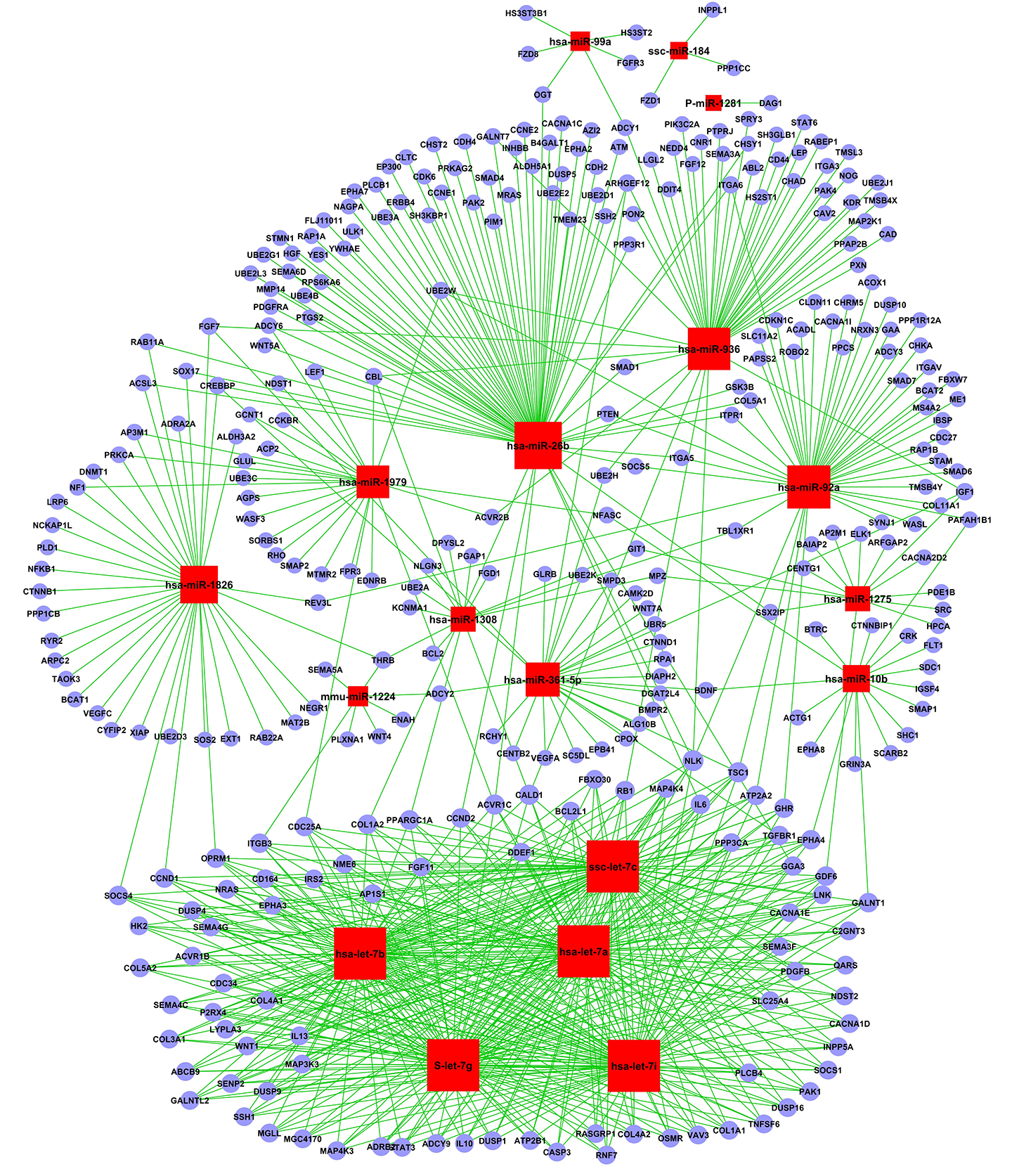

Supplement: Figure S1 — Potential targets prediction for differential expression miRNAs. (TIF) [file pone.0038640.s001.tif]

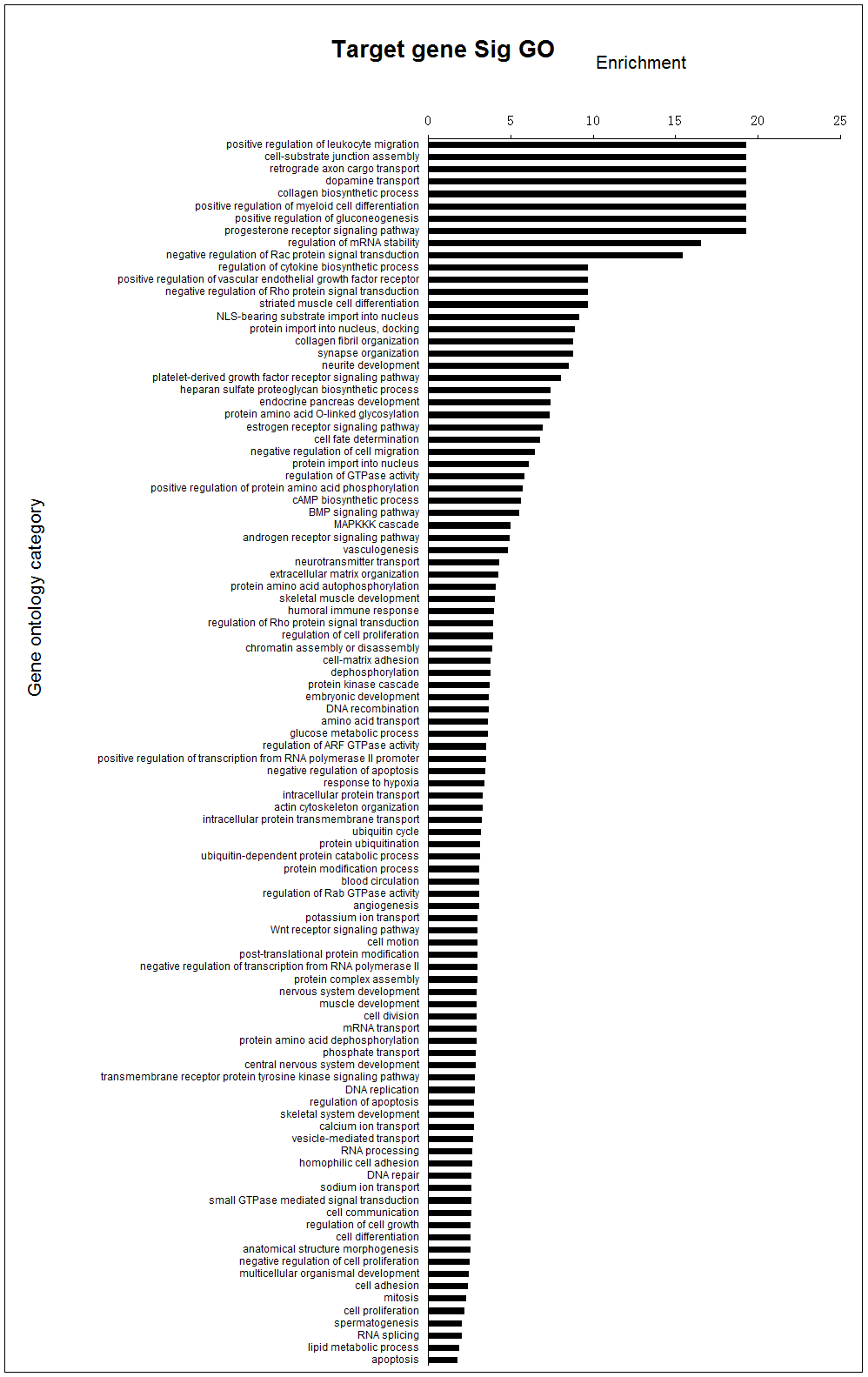

Supplement: Figure S2 — GO anslysis of potential targets. (TIF) [file pone.0038640.s002.tif]
